# Novel behaviour change frameworks for digital health interventions: A critical review

**DOI:** 10.1177/13591053231164499

**Published:** 2023-04-12

**Authors:** Melissa Pelly, Farhad Fatehi, Danny Liew, Antonio Verdejo-Garcia

**Affiliations:** 1 Monash University, Australia; 2 The University of Queensland, Australia; 3 The Alfred Hospital, Australia

**Keywords:** chronic illness, e-health, health behaviour, health psychology, intervention, models

## Abstract

Digital health interventions – interventions delivered over digital media to support the health of users – are becoming increasingly prevalent. Utilising an intervention development framework can increase the efficacy of digital interventions for health-related behaviours. This critical review aims to outline and review novel behaviour change frameworks that guide digital health intervention development. Our comprehensive search for preprints and publications used PubMed, PsycINFO, Scopus, Web of Science and the Open Science Framework repository. Articles were included if they: (1) were peer-reviewed; (2) proposed a behaviour change framework to guide digital health intervention development; (3) were written in English; (4) were published between 1/1/19 and 1/8/2021; and (5) were applicable to chronic diseases. Intervention development frameworks considered the user, intervention elements and theoretical foundations. However, the timing and policy of interventions are not consistently addressed across frameworks. Researchers should deeply consider the digital applicability of behaviour change frameworks to improve intervention success.

Potentially preventable chronic diseases linked to modifiable risk factors, including heart disease, respiratory disease and stroke, remain some of the highest causes of mortality in first-world countries (García et al., 2016; Khan et al., 2017). There is burgeoning interest and investment in developing accessible and scalable interventions, such as digital health interventions (DHI), that empower people to self-manage modifiable risk factors, including physical activity, diet and tobacco and alcohol use (García et al., 2016; Khan et al., 2017; Roberts et al., 2017). Chronic diseases are exacerbated by these risk factors and persist over a long period (Bernell and Howard, 2016), making continual (e.g. daily/weekly) support using DHI a promising approach to effective treatment. Covid-19 has changed the digital landscape of healthcare with an exponential rise in technologies to increase at-home care, such as remote health monitoring and interventions (Superina et al., 2022). This change makes it more important to have readily available systems to facilitate behaviour change in the digital space. Despite unprecedented interest in and availability of DHI, behaviours associated with risk factors (e.g. smoking, unhealthy eating, sedentarism) are deeply ingrained and difficult to change (Khan et al., 2017). This results in existing DHI facing difficulty in delivering effective and clinically meaningful behaviour change (Byrne, 2020; Roberts et al., 2017). Therefore, there is an onus on the healthcare system to escalate research that facilitates behaviour change using DHI for people with chronic conditions. This review will focus on leveraging behaviour change frameworks (tools that translate theory into practice) to guide the development of optimised DHI.

The potential of DHI to modify health risk-related behaviours relies on the effective application of behaviour change principles. Understanding the mechanisms of change (targets that play a role in initiating or maintaining behaviour change) is pivotal, with the National Institutes of Health (NIH) dedicating over a decade of research to the Science Of Behaviour Change (SOBC) project to uncover and elucidate the drivers of behaviour change (Nielsen et al., 2018). Without a clear understanding of these mechanisms of change, progress on effective behaviour change intervention development is limited (Nielsen et al., 2018). Nevertheless, behaviour change research continues to progress, with emerging, novel advances to behaviour change made each year. For instance, the behaviour change technique taxonomy consolidated 93 behaviour change techniques from 16 groupings to systematically guide the reporting of behaviour change techniques (Michie et al., 2013). Further, the interactive Theory and Techniques Tool through the Human Behaviour-Change project guides the decision on which behaviour change techniques to use through linkage to their mechanism of action (Michie et al., 2017a). Still, behaviour change techniques are best applied with guidance from appropriate behaviour change theories and/or frameworks.

Behaviour change theories represent an organised system that describes the behavioural principles used to modify attitudes and actions (Davis et al., 2015). Theories also specify the key ingredients that make behaviour change feasible, such as making the desired change simple and achievable (Taj et al., 2019). Complementing theories, behaviour change frameworks guide intervention development by proposing systematic steps to translate theory and behaviour change principles into practice (Taj et al., 2019). For the greatest chance at a comprehensive application of theory in a DHI, researchers should use a framework developed for the use of DHI, labelled in this paper as behaviour change frameworks for DHI. Behaviour change frameworks endeavour to systematise the application of behaviour change principles and techniques (Davis et al., 2015; Moller et al., 2017). However, Davis et al. (2015) found mixed results in the efficacy of applying theory to interventions, which they attributed to potentially misapplied theory and inappropriate choice of theory. Only 33% of DHI have used a framework to guide development (Taj et al., 2019). Whereas, almost double the rates (56.3%) of health behaviour interventions not specific to DHI reports applying theory (Prestwich et al., 2014). There is therefore a need to develop fine-grained analyses of behaviour change frameworks for DHI to improve framework development and selection, and ultimately intervention efficacy.

There is a need for behaviour change frameworks specific to DHI because they pose unique challenges and opportunities compared to non-digital interventions. Challenges for DHI include low levels of engagement and adherence (Torous et al., 2020), potentially due to the perceived impersonal nature of technology (O’Connor et al., 2016); and the fact that digital interventions often operate with little regulation (i.e. the mobile application industry) leading users to distrust technology (O’Connor et al., 2016). On the other hand, DHI poses considerable opportunities compared to non-digital approaches, including the ability to deliver accessible interventions every day, throughout the day, as needed (Nahum-Shani et al., 2015); and the ability to use algorithms to predict behaviour and adapt interventions accordingly to suit user context (Moller et al., 2017). Therefore, behaviour change frameworks for DHI can address the challenges of DHI explicitly while highlighting unique digital opportunities to enhance engagement that are not otherwise possible.

Five DHI domains are important for intervention success. These domains include accounting for: (1) individual *user* differences (Hekler et al., 2016; Medlock and Wyatt, 2019; Michie et al., 2017b; Pagoto and Bennett, 2013; Yardley et al., 2015; (2) *intervention* elements to drive behaviour change (Hekler et al., 2016; Medlock and Wyatt, 2019; Michie et al., 2017b; Morrison, 2015; Perski et al., 2017; (3) appropriate *timing* for optimal engagement, defined as the extent to which the intervention is used over time (Perski et al., 2017; Pinder et al., 2018; Yardley et al., 2016; (4) *theoretical foundations* which underlie the processes and basis of behaviour change (Moller et al., 2017; Morrison, 2015); and (5) *policy* requirements to improve the likelihood of commercialisation (Murray et al., 2016). Adhering to policy ensures efficient use of time and financial resources while reducing the health-related opportunity cost of potential delays in launching an intervention aimed to promote health behaviour (Murray et al., 2016). Because DHI is a relatively emerging field for healthcare delivery, likely many behaviour change frameworks for DHI do not yet explicitly address these domains. Therefore, further guidance on incorporating these domains when assessing and developing behaviour change frameworks for DHI is needed. Theoretically, an intervention modelled using a framework that incorporates a high amount of these elements will have a greater likelihood of effective behaviour change.

Prior reviews conducted on DHI frameworks tend to primarily identify or categorise DHI frameworks. A scoping review by Taj et al. (2019) identified 15 frameworks that have been used in the context of DHI behaviour change. Meanwhile, the Carbonnel and Ninot (2019) narrative review categorised frameworks used in DHI based on framework structure, such as whether the framework had a linear progression of steps. Prior to these reviews, to our knowledge, no review had been published on behaviour change frameworks for DHI. Since 2019, research has expanded to pursue frameworks specifically developed for DHI. In 2020, a scoping review protocol was developed for frameworks that implemented or evaluated digital health (Soobiah et al., 2020). Another scoping review explored the makeup and outcomes of DHI frameworks for chronic diseases (Bashi et al., 2020). While these scoping reviews have highlighted different development methods, compositions and mechanisms of frameworks, we intend to focus on an overall analysis of how extensively contemporary behaviour change frameworks for DHI suit current DHI necessities.

The heightened focus on behaviour change frameworks for DHI in recent years informs the scope of our study. Researchers should keep aware of new and revolutionary behaviour change frameworks that can guide intervention development. This ensures novel, cutting-edge research is informing the intervention and advancing the research field. Taj et al. (2019) illustrated a relatively low number of behaviour change frameworks for DHI (15 frameworks identified over the previous two decades). We, therefore, aim to review behaviour change frameworks for DHI published from 2019 to inform researchers in this field of novel frameworks they may not be aware of. Our objective is to identify, categorise and review frameworks proposed in published articles and preprints from 2019. The primary aim of this study is to critically review and evaluate the most recently proposed behaviour change frameworks for the design and implementation of digital health for chronic disease. However, we acknowledge there are other aspects of intervention development that are unrelated to the digital medium that can be important when selecting which frameworks to use, for example, the resources, time, and competencies of the research team. For those non-digital aspects, we refer readers to competency and resource materials outside of this review, such as Dixon and Johnston (2021) and Sumner et al. (2018).

## Methods

### Information sources and search strategy

The review protocol was not registered. We conducted a comprehensive search to identify theoretical frameworks for digital behaviour change using PubMed, PsycINFO, Scopus and Web of Science, and included preprint publications using the Open Science Framework repository. Two reviewers collaborated on developing a search strategy for relevant publications. The primary electronic search was conducted in August 2021. The search focussed on (1) digital health interventions, (2) behaviour change and (3) framework development. The search terms allowed the search to remain broad (e.g. did not specify chronic disease) to ensure articles were not incorrectly excluded from the output. The search was limited to articles written in English and published between 01/01/2019 and 01/082021. The search terms are included in Appendix A.

Articles were included if they: (1) were peer-reviewed; (2) proposed a behaviour change framework to guide digital health development; (3) were written in English; (4) were published between 1/1/2019 and 1/8/2021; and (5) were applicable to chronic disease health behaviour (excluding negative health behaviours that do not persist over time, e.g. vaccine promotion). Articles were excluded if they did not result in the proposal of a framework detailing behaviour change concepts for DHI.

### Study selection

**Figure 1.**
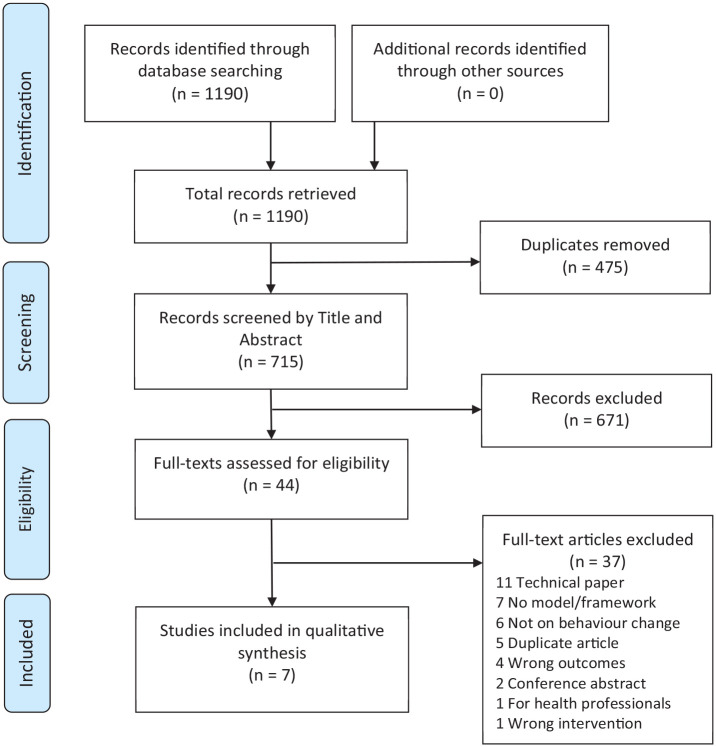
The process of identifying articles for behaviour change frameworks for digital health proposed from January 2019 to August 2021.

The search results were imported into Endnote from each database and duplicates were removed. The remaining articles were imported into Covidence (www.covidence.org). Two researchers independently screened all articles based on their title and abstract to determine eligibility. The researchers consulted on all conflicts using a conservative approach with most articles being included in the next stage to reduce the risk of bias. The two researchers then read the full text of all articles and independently assessed each one based on the inclusion and exclusion criteria. Conflicts were resolved by consensus: the two assessors met and explained their reasoning for including/excluding the article regarding specific inclusion and exclusion criteria while looking through the article together until a final decision was agreed on. Technical papers were the most common reason for exclusion, that is, the framework focusing on the technical wireframe rather than behaviour change. Figure 1 illustrates a breakdown of the search process. A systematic approach to comprehensively screen for relevant conceptual frameworks has been applied in this critical review (Linares-Espinós et al., 2018). From here, we have focussed on the critical appraisal of the theoretical landscape of behaviour change frameworks for DHI (Carnwell and Daly, 2001; Cronin et al., 2013).

### Data collection and synthesis

After title/abstract screening and the inspection of full texts, a summary of each identified framework was made. This included the author/s, a summary of the article, the title, the country of publication, the aim of the article, the study design and the published date were created in a spreadsheet using Microsoft Excel and circulated between all authors. There was no missing information.

To aid our critical analysis of the frameworks, we created a second spreadsheet in Microsoft Excel detailing how each framework addressed five primary domains to aid digital behaviour change intervention development: the users (Medlock and Wyatt, 2019); intervention (Medlock and Wyatt, 2019); timing (Pinder et al., 2018; Yardley et al., 2016); theoretical foundation (Naslund et al., 2017); and policy of intervention implementation (Murray et al., 2016). Table 1 outlines these evaluation criteria with 17 items that encapsulate different strands of evidence from DHI literature, assessed under five domains. Each item was rated ‘yes’, ‘somewhat’ or ‘no’. Information missing from these domains was deemed to not be present in the framework. Although these criteria are not only relevant for DHI but also for behaviour change interventions more broadly, the criteria have specific implications in the context of DHI. For instance, DHI allows for fine-grained, personalised customisation of the intervention to each user’s preferences, while adapting to the user’s day-to-day context with tailored interventions (user and intervention domains). DHI can deliver frequent interventions consistently throughout the day and readily adapt the dosage requirements of the intervention on a day-to-day basis based on user behaviour (timing domain). Unlike in-person interventions, DHI can use algorithms informed by previous behavioural data to predict future behaviour and dynamically tailor the intervention using theory-based behaviour change techniques adapted for technology (theoretical foundations domain). Lastly, DHI modalities have different regulations than in-person interventions; DHI policy requirements should not be treated as synonymous with typical in-person behaviour change interventions (policy domain). From here, we compared frameworks based on these DHI domains and holistically reviewed the current state of novel behaviour change frameworks for DHI.

**Table 1.** The evaluation criteria for behaviour change frameworks for digital health.

Domains	Items
User	Does the framework: 1. Specify/guide on the *target groups* for the intervention? 2. Consider the *culture* of target groups? 3. Consider the *context* for behaviour change interventions? 4. Allow users to *customise* the contents? 5. Consider *social support* as part of the intervention?
Intervention	Does the framework: 6. Guide how to develop behaviour change *techniques*? 7. Allow interventions to be *tailored* to the target population/user? 8. Allow *adaptive* contents? 9. Inform the best *form* of content delivery?
Timing	Does the framework: 10. Specify the *time* that the interventions should be delivered? 11. Specify the *frequency* of the intervention? 12. Guide the *duration* of the intervention? 13. Guide how to determine the correct *dose* of the intervention?
Theoretical Foundations	Does the framework: 14. Allow the development of *theory-based* techniques? 15. Allow the *prediction* of the users’ behaviour?
Policy	Does the framework: 16. Indicate if any *policy* change is required for the success of the interventions? 17. Indicate if any *regulation* is needed for the implementation of the intervention beyond the research phase?

## Results

The search yielded seven novel behaviour change frameworks for DHI which met eligibility criteria. The structure of these frameworks falls into one of two categories. The first category is frameworks that focus on the mechanisms of behaviour change, that is, what the intervention should address to change behaviour. We will refer to these as ‘Mechanistic frameworks’. The second category is frameworks that focus on the steps behind digital behaviour change development from inception to implementation. We will refer to these as ‘Stepwise frameworks’. This categorisation can also be roughly applied to behaviour change frameworks for DHI outside of this review. The decision on which category of framework to use may differ between research teams, for example, development and commercialisation experts may choose a Mechanistic framework to fill the knowledge gap concerning mechanisms of action for behaviour change. To help researchers compare these frameworks, we will now detail the core principles of each framework based on their framework structure. Table 2 outlines the degree to which each framework satisfied the evaluation criteria domains. Frameworks used in this review can be found online using a database search.

**Table 2.** Novel behaviour change frameworks based on an evaluation criteria.

Framework structure	Framework name	User	Intervention	Timing	Theoretical foundation	Policy
Mechanistic	ADM	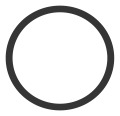	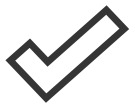	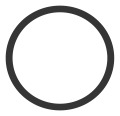	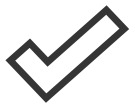	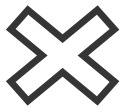
	ABC	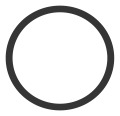	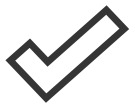	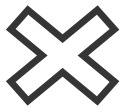	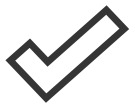	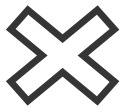
	EOI	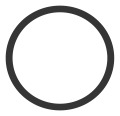	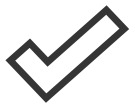	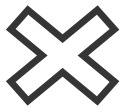	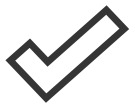	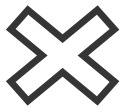
Stepwise	TUDER	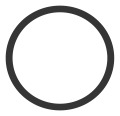	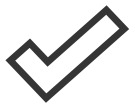	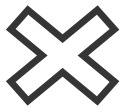	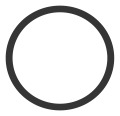	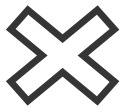
	IFDBCI	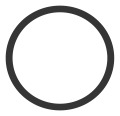	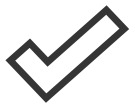	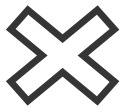	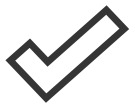	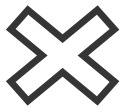
	UCAI	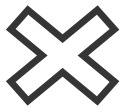	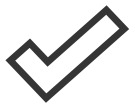	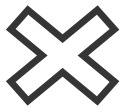	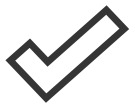	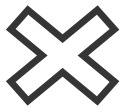
	PMHA	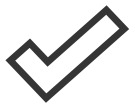 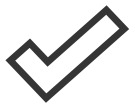	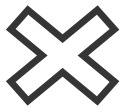	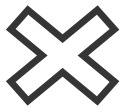	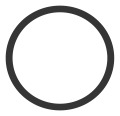	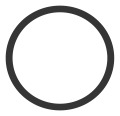

The ticks indicate the framework adequately or highly addressed the domain. The circles indicate the framework somewhat addressed the domain. The crosses indicate the framework did not address the domain adequately. These scorings are based on the average of each domain. The Mechanistic frameworks include: ABC: Adaptive Behavioural Components model; ADM: Adaptive Decision Making framework; EOI: Effort-Optimised Intervention Model. The Stepwise frameworks include: TUDER: Targeting, Understanding, Designing, Evaluating and Refining framework; IFDBCI: Iterative Framework for Digital Behaviour Change Interventions; UCAI: User-Centric Adaptive Intervention model; PMHA: Persuasive Mobile Health Application model.

### Mechanistic frameworks

#### (1) Adaptive Decision Making framework

The Zhang et al. (2021) Adaptive Decision Making framework focuses on the temporal manner in which interventions are implemented. It suggests interventions can be delivered at different times based on two aspects. The first temporal aspect is lifestyle behaviours, represented by individual daily decisions (action-level), or broader behavioural episodes (reflection-level) which represent decision-making over a period of time. The second aspect is the dynamic relationship between behaviours and cognitive constructs, such as knowledge of risk factors. Interventions should map action-level and reflection-level processes about learning or decision-making processes so that learning can be action-level or reflection-level, and decision-making can be action-level or reflection-level.

The Adaptive Decision Making framework extensively addresses intervention domains, with detailed guidance of behaviour change techniques, and tailored and adaptive elements. It also moderately addresses theoretical foundations with some degree of behaviour prediction, while moderately addressing user characteristics. The framework somewhat captures timing with a strong focus on temporality but does not capture dosage or duration requirements for an effective intervention, that is, how intensively, and to what degree do each reflection- and action-level considerations need to be addressed for an effective intervention? The framework does not capture policy considerations.

#### (2) Adaptive Behavioural Components model

This model has a high focus on tailoring digital health to specific behavioural characteristics, population demographics, technology usage and individual needs (Young, 2020). Firstly, the model implements a range of tested behaviour change principles and methods, such as making the desired behaviour easy to engage in. Secondly, problem-focused components tailor the intervention to specific behaviours using reminders, tracking, and calendars. Next, tailoring to population (demographic characteristics), social (community support) and behavioural (context of behaviour, such as work-life) components are considered to improve intervention efficacy. Individual factors such as personality are explained to influence likelihood to engage with an intervention. Lastly, long-term consideration of technology-related trends is critical to the success of a digital intervention.

Overall, the Adaptive Behavioural Components model emphasises research-supported personalisation and tailoring to promote greater uptake of the intervention. This shows extensive consideration for intervention domains and theoretical foundations and moderate levels of consideration for user domains. However, there is little guidance on the order or timing of applying these elements into an intervention or how a researcher should navigate policy when bringing the intervention to market.

#### (3) Effort-Optimised Intervention model

This model centres around exploring and optimising the user’s effort to partake in the targeted behaviour (Baumel and Muench, 2021). The model proposes a three-phase approach to develop an effort-optimised intervention. The first phase is nurturing salience, the degree to which the behaviour is considered important. Promoting task availability, displaying an actionable script to follow, implementing rewards and optimising novelty can nurture salience. The next phase is to make the therapeutic activities as effortless as possible. Lastly, the model aims to translate their effort into a commitment which can be achieved by reflecting on the user’s past efforts in a meaningful way.

Overall, the Effort-Optimised Intervention Model is well poised to promote both short-term and long-term behaviour change. It facilitates the achievement and reinforcement of target behaviours through highly specified intervention domains based on extensive theoretical foundations. However, it only somewhat addresses user characteristics, with high consideration of the users’ context and some consideration of social support. Timing was somewhat addressed, while the frequency, duration and dosage of long-term engagement were not addressed. Policy was not addressed.

### Stepwise frameworks

#### (4) Targeting, understanding, designing, evaluating and refining framework

This holistic and iterative framework proposes four sequential steps, that is, targeting, understanding, designing and evaluating/refining (Wang et al., 2019). (1) Targeting and learning about the specific population, health problem and behaviour leads to a better understanding of the requirements of the intervention. (2) Researching behavioural theories and models will help to Understand the behaviour and strategies to elicit behaviour change. (3) The Design of the intervention involves optimising the workflow by incorporating time-based or task-based interventions rather than developing a static intervention. (4) Finally, the intervention should be Evaluated and Refined based on research trends and pilot studies.

Overall, the framework provides a comprehensive pipeline for intervention development, with some reference to theoretical foundations using intervention taxonomy research and guidelines to deliver intervention domains. The framework somewhat considers the user domains, including the target group, user context and social support, but minimally addresses the timing of interventions. Policy was not addressed.

#### (5) Iterative Framework for Digital Behaviour Change Interventions

The Iterative Framework for Digital Behaviour Change Interventions aims to utilise up-to-date technology to develop an intervention best tailored to individual needs in five stages (Sucala et al., 2020). (1) The Pre-define phase involves developing an understanding of the population, targeted health outcomes and context of the intervention. (2) Defining involves considering the intervention and conceptual model using previous research and expert advice. (3) Designing involves both the front-end interface (creation of wireframes) and back-end systems (design of system architecture) for user testing. (4) Developing involves incorporating user feedback into a minimum viable intervention for a test environment and then in real-world settings. (5) Deploying involves disseminating the intervention in beta environments and then in real-world settings.

Overall, the Iterative Framework for Digital Behaviour Change Interventions is an attractive framework to detail the transformation from conceptual design to product, with substantial theoretical foundations outlining considerable advice in the intervention domain (yet little advice on specific behaviour change techniques). The framework moderately addresses user domains, except for the specification of a target group, but minimally addresses policy and timing of interventions.

#### (6) User-Centric Adaptive Intervention model

This model has a high emphasis on leveraging technology for behaviour change using four overarching stages (Bilal et al., 2020). (1) Behaviour Quantification involves assigning a value to behaviours based on expected years of life lost to identify integral behaviours to target. (2) Behaviour-Context Mapping involves mapping healthy behaviours onto different behaviour contexts, such as those contemplating versus actively engaged in behaviour change. (3) Intervention Selection has been ramified into two primary styles: authoritative, such as explicit direction to the user; and facilitative, such as reducing negative self-doubt. (4) Feedback evaluation, involves collecting explicit and implicit (objective data) feedback from users after deploying a trial.

Overall, the User-Centric Adaptive Intervention model’s focus on targeting behaviours would be useful for those aiming to narrow their intervention’s focus. This shows a high-level consideration for the intervention domain, coupled with extensive consideration of theoretical foundations. The framework is complex and could be difficult to implement without ample time, expertise and resources. Additionally, the timing and users were minimally addressed, and policy considerations were not addressed.

#### (7) Persuasive Mobile Health Application model

The Persuasive Mobile Health Application model synergistically combines two well-documented frameworks, the Persuasive System Design (Oinas-Kukkonen and Harjumaa, 2009) and Behaviour Change Wheel (Michie et al., 2011), into one holistic approach (Sari et al., 2020). After defining the problem in behavioural terms and selecting the ideal targeted behaviour, the Persuasive Mobile Health Application model introduces the Behaviour Change Wheel’s capability, opportunity and motivation. The taxonomy for behaviour change techniques is then mapped onto identified barriers for behaviour change. Lastly, the behaviour change techniques that have been selected will inform which Persuasive System Design features will be used.

Overall, the Persuasive Mobile Health Application model displays a simple methodology to incorporate two well-known frameworks into one synergistic framework. The model extensively considered policy (due to the usage of the Behaviour Change Wheel) and user domains (save for cultural considerations). However, theoretical foundations were only somewhat addressed, with no ability to make predictions for future behaviour, and the intervention and timing domains were minimally addressed.

## Discussion

Behaviour change frameworks for DHI each have unique strengths and weaknesses that can facilitate or hinder intervention development. From here, based on the evaluation criteria, researchers will be guided on which domains were common in the reviewed frameworks to identify a gap that should be addressed in future framework proposals.

Overall, the theoretical foundations and intervention domains of DHI behaviour change frameworks were well-addressed. The high attention to detail in theory is not surprising considering the vast body of existing research on behaviour change (Davis et al., 2015), with the Persuasive System Design (Oinas-Kukkonen and Harjumaa, 2009), Behaviour Change Wheel (Michie et al., 2011), Social Cognitive Theory (Bandura, 1998) and Theory of Planned Behaviour (Ajzen, 1991) being commonly cited amongst the reviewed frameworks. All frameworks extensively formed linkages with the theory underpinning development, with four of seven frameworks addressing the theoretical foundations and prediction of behaviour to a very high degree. Similarly, the intervention domain was greatly addressed with every reviewed framework informing on behaviour change techniques – the cornerstones of effective behaviour change (Morrison, 2015). Frameworks also emphasise tailoring to the user and using adaptive contents to provide relevant and real-time interventions (Hekler et al., 2016; Medlock and Wyatt, 2019). Overall, this review shows the theoretical foundations and intervention domains of contemporary frameworks have been well addressed.

However, the user domain was only somewhat covered by the reviewed frameworks. While the context of interventions and social support were well addressed (Hekler et al., 2016; Michie et al., 2017b), the culture of participants was not covered extensively by most frameworks. Healthcare has the intention to ‘do no harm’, but harm is an unintended and unseen consequence if the accessibility and usability of DHI by different cultures are not considered (Michie et al., 2017b). Some frameworks did not guide choosing a target group for the intervention, yet frameworks may not be ideal for all target groups (Medlock and Wyatt, 2019). For example, behaviours that are associated with more deeply engrained conditions, such as alcohol use disorder, may require more comprehensive and specific frameworks compared to behaviours that are comparatively straightforward to modify. Likewise, some frameworks did not advise on user customisation of the intervention, which could reduce engagement and adherence (Medlock and Wyatt, 2019; Yardley et al., 2016). This review shows contemporary frameworks somewhat address the user of the intervention, but newly proposed frameworks would benefit from incorporating this domain further.

Lastly, most frameworks lacked guidance on the timing and policy domains. Timing is necessary for DHI where interventions can be delivered at any hour of the day (Perski et al., 2017; Pinder et al., 2018; Yardley et al., 2016). There was some consideration for the exact time that interventions should be delivered, but almost no consideration of the frequency, duration or dose of interventions, exacerbating the issue of low engagement (Perski et al., 2017). ‘Just-in-time’ interventions can address this concern by adapting over time to changing circumstances and delivering interventions at the correct time, frequency, duration and dosage for each user’s lifestyle (Nahum-Shani et al., 2015; Pinder et al., 2018). In addition, the policy that underpins interventions was not considered well, with only two Stepwise frameworks mentioning these. Without policy consideration, difficult, large-scale changes may be needed after product development (Michie et al., 2017b; Murray et al., 2016). Intervention researchers should explicitly outline any relevant requirements or guidelines they have addressed (Michie et al., 2017b), such as good practice guidelines on how to report technical features which otherwise may be difficult to interpret by policy-makers (Agarwal et al., 2016; Eysenbach, 2011). Therefore, this review shows the lack of timing and policy considerations could be a detriment to intervention efficacy and commercialisation; future framework development should incorporate these domains.

Researchers involved in framework development could use our findings to improve the targeting of the identified unmet domains, that is, the user, timing and policy domains. The Persuasive Mobile Health Applications framework is a good example of incorporating the information derived from multiple frameworks into one easily digestible framework. This allows researchers to harness the unique benefits of multiple impactful frameworks to achieve a greater likelihood of addressing more DHI domains. For instance, the Persuasive Mobile Health Applications framework usage of the Behaviour Change Wheel incorporates unique guidance on the policy and regulations of interventions into a framework that had not otherwise considered this. Our findings regarding the strengths and weaknesses of different frameworks could be used to inform the development of more holistic approaches that integrate specific elements of existing models in a time-efficient manner that better suits the time demands of the rapidly evolving landscape of digital health. Additionally, there is little cross-over between the Mechanistic and Stepwise categories, forcing intervention developers to choose between these categories, or use multiple frameworks to be guided on both categories (requiring excessive resources). Neither outcome is ideal. Future frameworks may benefit from incorporating both category types as a more holistic guide to intervention development. Covering both Mechanistic and Stepwise elements may result in a framework that addresses more DHI domains, ultimately leading to a greater likelihood of intervention success.

### Strengths and limitations

The strengths of this review lie in the comprehensive search that was conducted, and the systematic approach towards the screening and selection of the eligible articles. Both new and preprint publications were included to ensure we have accounted for all contemporary behaviour change frameworks for DHI. We comprehensively described each framework; understanding the make-up of the included frameworks gives the reader context when interpreting our conclusions and informs the development of the future proposed frameworks.

The current review has some limitations which are important to address. Firstly, while we conducted a comprehensive search on various databases for new behaviour change frameworks, we potentially missed a newly proposed framework. Articles could have used keywords that were not searched, or failed to mention the framework in their abstract resulting in exclusion at the title and abstract screening. Likewise, there may be other DHI requirements needed for an effective behaviour change intervention that we did not consider. We advise intervention developers to conduct further research to have the best chance at intervention success. Additionally, the criteria to guide our evaluation of the behaviour change frameworks for DHI have not been validated. However, the criteria were solely developed to supplement the critique of frameworks and provide structure to our analysis.

## Conclusion

In conclusion, we reviewed seven novel frameworks that researchers should consider when developing a behaviour change intervention for DHI. We aim to inform the next generation of frameworks on the DHI domains that have room to improve, that is, the user, timing and policy domains. This review ultimately informs the development of well-supported and pioneering frameworks which can change the landscape for digital behaviour change interventions as we know it.

## Supplemental Material

sj-csv-3-hpq-10.1177_13591053231164499 – Supplemental material for Novel behaviour change frameworks for digital health interventions: A critical review Click here for additional data file. Supplemental material, sj-csv-3-hpq-10.1177_13591053231164499 for Novel behaviour change frameworks for digital health interventions: A critical review by Melissa Pelly, Farhad Fatehi, Danny Liew and Antonio Verdejo-Garcia in Journal of Health Psychology

sj-csv-4-hpq-10.1177_13591053231164499 – Supplemental material for Novel behaviour change frameworks for digital health interventions: A critical review Click here for additional data file. Supplemental material, sj-csv-4-hpq-10.1177_13591053231164499 for Novel behaviour change frameworks for digital health interventions: A critical review by Melissa Pelly, Farhad Fatehi, Danny Liew and Antonio Verdejo-Garcia in Journal of Health Psychology

sj-docx-1-hpq-10.1177_13591053231164499 – Supplemental material for Novel behaviour change frameworks for digital health interventions: A critical review Click here for additional data file. Supplemental material, sj-docx-1-hpq-10.1177_13591053231164499 for Novel behaviour change frameworks for digital health interventions: A critical review by Melissa Pelly, Farhad Fatehi, Danny Liew and Antonio Verdejo-Garcia in Journal of Health Psychology

sj-docx-2-hpq-10.1177_13591053231164499 – Supplemental material for Novel behaviour change frameworks for digital health interventions: A critical review Click here for additional data file. Supplemental material, sj-docx-2-hpq-10.1177_13591053231164499 for Novel behaviour change frameworks for digital health interventions: A critical review by Melissa Pelly, Farhad Fatehi, Danny Liew and Antonio Verdejo-Garcia in Journal of Health Psychology
