# Supplementary material for: Novel behaviour change frameworks for digital health interventions: A critical review
Source: J Health Psychol. 2023 Apr 12;28(10):970–83. doi: 10.1177/13591053231164499 (PMC10466959; doi:10.1177/13591053231164499)
Supplement: sj-docx-1-hpq-10.1177_13591053231164499 – Supplemental material for Novel behaviour change frameworks for digital health interventions: A critical review [file sj-docx-1-hpq-10.1177_13591053231164499.docx]

Appendix A: Literature Search Queries

The search query used in each database broadly depicted:

1) digital health,

2) behaviour change,

3) frameworks,

4) Publication between 2019/1/1 and 2021/08/1,

5) English language.

Searches conducted in each database comprised all of the below constructs combined with AND:

("digital" OR "ehealth" OR "electronic health" OR "mhealth" OR "mobile health" OR "mobile-based" OR "telehealth" OR “telemedicine” OR "web-based" OR “Internet”)

AND

("behaviour change" OR "behavior change")

AND

("framework" OR "model")

Filters:

Publication date: 1/1/2019 until 1/08/2021

Language: English
