# Supplementary material for: Novel behaviour change frameworks for digital health interventions: A critical review
Source: J Health Psychol. 2023 Apr 12;28(10):970–83. doi: 10.1177/13591053231164499 (PMC10466959; doi:10.1177/13591053231164499)
Supplement: sj-docx-2-hpq-10.1177_13591053231164499 – Supplemental material for Novel behaviour change frameworks for digital health interventions: A critical review [file sj-docx-2-hpq-10.1177_13591053231164499.docx]

Appendix B: Explanatory Memo Statement

Supplementary files in this manuscript include:

Appendix A:

This outlines the queries used for the article search conducted.

Supplementary Material A:

This includes for each reviewed framework the: Covidence number, study ID, published date, reviewer name, study ID number, study design, country in which the study conducted, title, aim of study and a one sentence summary of framework.

Supplementary Material B:

A spreadsheet including data the authors have generated from the papers included in this review. Two authors collaborated on the development of this spreadsheet. The first column identifies the framework category (Mechanistic or Stepwise). The second column identifies the article name. The third column identifies the framework name.

From here, columns D-X depict each criteria item and the totals for each domain. The numerical values were assigned through careful reading of the article proposing the framework. A consensus between two authors was made on the scoring of these criteria items. Scorings of 0 means the criteria was not mentioned. Scorings of 1 means the criteria was somewhat addressed. Scorings of 2 means the criteria was addressed well.

Columns AA-AC show the overall scores for each framework across all domains.

As a summary, the frameworks were each listed in rows 21-28 with their overall score. Rows 21-28 also contains the domain scoring, with the average scoring of each domain broken down by the framework category.

Software used:

The Covidence system was used for article screening. No additional software was used for the creation of this manuscript.
